# Potential Modes of Intercellular α-Synuclein Transmission

**DOI:** 10.3390/ijms18020469

**Published:** 2017-02-22

**Authors:** Dario Valdinocci, Rowan A. W. Radford, Sue Maye Siow, Roger S. Chung, Dean L. Pountney

**Affiliations:** 1 Menzies Health Institute Queensland, Griffith University, Gold Coast 4222, Australia; dario.valdinocci@griffithuni.edu.au (D.V.); suemaye.siow@griffithuni.edu.au (S.M.S.); 2 Department of Biomedical Sciences, Faculty of Medical and Health Sciences, Macquarie University, Sydney 2109, Australia; rowan.radford@students.mq.edu.au (R.A.W.R.); roger.chung@mq.edu.au (R.S.C.)

**Keywords:** α-synuclein, Parkinson’s disease, multiple system atrophy, dementia with Lewy bodies, exosome, tunneling nanotube, gliosis, glymphatic

## Abstract

Intracellular aggregates of the α-synuclein protein result in cell loss and dysfunction in Parkinson’s disease and atypical Parkinsonism, such as multiple system atrophy and dementia with Lewy bodies. Each of these neurodegenerative conditions, known collectively as α-synucleinopathies, may be characterized by a different suite of molecular triggers that initiate pathogenesis. The mechanisms whereby α-synuclein aggregates mediate cytotoxicity also remain to be fully elucidated. However, recent studies have implicated the cell-to-cell spread of α-synuclein as the major mode of disease propagation between brain regions during disease progression. Here, we review the current evidence for different modes of α-synuclein cellular release, movement and uptake, including exocytosis, exosomes, tunneling nanotubes, glymphatic flow and endocytosis. A more detailed understanding of the major modes by which α-synuclein pathology spreads throughout the brain may provide new targets for therapies that halt the progression of disease.

## 1. α-Synuclein

α-Synuclein (α-syn) is a 140 amino acid, soluble protein found predominantly within the central nervous system (CNS), and is enriched in the peripheral nervous system and circulating erythrocytes [1,2,3]. The normal role of α-syn remains unclear, however high concentrations of the protein exist within neuronal presynaptic terminals, indicating potential function(s) in synaptic transmission [4]. Multiple specific roles have been proposed for α-syn, including soluble NSF attachment protein receptor (SNARE) protein regulation, production of dopamine and regulation of synaptic vesicle recycling [5,6,7,8,9]. Contention exists as to the normal cellular conformation of α-syn. Recombinant α-syn exists as an unfolded monomer, whereas recent evidence shows that extraction from eukaryotic sources, such as erythrocytes or human brain tissue, produced α-syn multimers, mostly tetramers, with α-helical content [10,11,12]. Burre et al and Theillet et al., provide evidence that α-syn normally exists largely as an unfolded monomer in the free cytosolic form whilst adopting an α-helical conformation once bound or interacting with lipid membranes [2,13].

## 2. α-Synuclein in Disease

Intracellular inclusion bodies comprised primarily of misfolded and aggregated α-syn are the neuropathological hallmark of a group of neurodegenerative diseases with overlapping complex clinical phenotypes. These are known collectively as α-synucleinopathies and include Parkinson’s disease (PD), multiple system atrophy (MSA) and dementia with Lewy bodies (DLB) [14]. PD patients develop characteristic motor dysfunction such as bradykinesia, tremor, rigidity and postural instability which are collectively known as Parkinsonism. As the disease progresses, patients often develop depression, anxiety and dementia in advanced stages [15,16]. DLB patients present with Parkinsonism and cognitive impairment such as hallucinations, fluctuations in alertness and short-term memory with a relatively rapid progression and decline [17]. MSA is classified into two subcategories, based on α-syn distribution, pattern of spread, neurodegeneration and clinical phenotypes. MSA-P (Parkinsonism) patients typically display a combination of parkinsonian-like symptoms, such as tremors and bradykinesia, whilst MSA-C (cerebellar ataxia) cases present with signs of ataxia and gait disturbance [18,19]. Both forms also display autonomic dysfunction in addition to other symptoms. α-Synucleinopathies exhibit overlap with regard to brain regions affected, with all three disorders displaying neuronal loss, particularly of dopaminergic neurons, within the substantia nigra in the midbrain as well as the basal ganglia [19,20]. Differences in disease duration also exist between the three diseases, with MSA patients surviving for around 8 years after symptoms appear, whilst PD and DLB typically survive more than 10 years [21,22,23].

In addition to neuronal loss, α-synucleinopathies each display characteristic inclusions, with the predominant types in PD and DLB known as the Lewy body (LB) and the Lewy neurite (LN). LBs and LNs both share similarities, such as their composition of filamentous α-syn, ubiquitin and neurofilament and are found in surviving neurons upon autopsy [24,25,26,27]. However, these inclusions have distinctly different morphologies, with LBs being largely spherical whilst LNs have rod- or snake-like morphologies [26]. Work by Kanazawa et al., using a three-dimensional (3D) reconstruction of confocal images revealed that both LB and LN share the same structural components of an inner filamentous α-syn and ubiquitin layer coated by neurofilament [28], also finding LB/LN intermediary-like structures sharing the same characteristics. This suggests that LNs may be an earlier LB stage, thus explaining the abundance of these inclusions together in affected brain regions. MSA inclusion bodies, termed glial cytoplasmic inclusions (GCIs), share similarities to LBs and LNs, containing filamentous α-syn and ubiquitin, however, also contain αB-crystallin and tau [29,30]. GCIs have a triangular or sickle-like morphology [29,31] and occur predominantly in oligodendrocytes [32]. Although oligodendrocyte lineages are known to express α-syn mRNA at low levels both normally and in MSA, α-syn is expressed primarily in neurons, implicating uptake as the main source of α-syn in GCIs [33,34].

Although the majority of cases of α-synucleinopathy are sporadic, rare missense mutations in the α-syn gene (*SNCA*) have been found to cause familial PD phenotypes and α-syn pathology with some variants also displaying pathology similar to MSA [35,36]. Gene duplications and triplications have also been reported resulting in increased α-syn synthesis and PD phenotypes [37]. Furthermore, genome wide association studies have highlighted *SNCA* as one of the strongest risk loci for developing PD, strengthening the link to α-syn as a causative agent in α-synucleinopathies [38,39,40]. The intracellular aggregation of α-syn can be triggered by a wide range of stimuli, including raised metal ion concentrations, oxidative stress and post-translational modifications, such as phosphorylation at Serine 129 and Serine 87 or other modifications such as nitration [41,42,43]. Phosphorylation at Serine 129 can be associated with an insoluble, oligomeric β-sheet conformation and has been shown to be the dominant modification in MSA and DLB [44,45]. Oligomeric α-syn is then thought to aggregate into fibrils, the characteristic form of α-syn observed in diseases such as PD. These fibrils together with a multitude of other proteins form the pathological inclusion bodies. The central hydrophobic region of α-syn termed non-Aβ Component (NAC), comprising residues 61–95, is the region susceptible to β-sheet formation [46]. It has been described recently that α-syn can form strains in vitro other than fibrils which display different binding, penetration and toxic characteristics as well as a different structure, such as α-syn ribbons [47]. Whilst ribbons have not yet been detected in humans, various strains of oligomers have been observed [48,49]. These different strain types may account for some of the differences observed in different α-synucleinopathies, such as inclusion body morphology or cell types affected.

## 3. α-Synuclein and Disease Spread

The current model of tissue spread of α-syn pathology, devised by Braak et al., in 2003 proposed that LB in sporadic PD originate within the olfactory bulb and medulla oblongata and later spread to the substantia nigra, midbrain and basal ganglia [20]. In MSA-P, degeneration occurs within the nigrostriatal region due to appearance of α-syn in the substantia nigra and putamen, later spreading to the frontal cortex [50]. MSA-C has recently been proposed to have a 4-phase stage pathology, whereby it begins in the cerebellar subcortical white matter and olivo-cerebellar fibres within the medulla, later spreading to the basal ganglia, neocortex, amygdala and hippocampus [51]. A spatial relationship between GCIs and myelinated axons was also observed, revealing that GCIs occur largely in myelinating oligodendrocytes. Consensus guidelines of the DLB Consortium aim to classify DLB pathology via LB presence and density in specific brain regions in conjunction with the presence of Alzheimer’s-like degeneration. Typical DLB classifications have intermediate to high LB load within the neocortical regions with an intermediate Alzheimer’s-like degeneration [52]. The presence of Alzheimer’s pathology is also part of the diagnostic criteria for DLB. Experiments with in vivo animal models have revealed the cell-to-cell transmission of pathological α-syn. Different synthetic strains (oligomers, ribbons and fibrils) are observed to have variable pathogenicity and uptake when systemically or locally administered into mice [53]. Luk et al. (2012) showed that single intrastriatal inoculation of preformed fibrils into non-transgenic mouse brains caused LB appearance and PD-like symptoms [54]. Using longitudinal in vivo multiphoton imaging, Ostenberg et al. (2015) demonstrated that inoculation of synthetic α-syn fibrils induced aggregation, inclusion formation and subsequent death of a large proportion of neurons harboring inclusions [55]. Other studies have shown similar results, with α-syn spread found to be a potential factor leading to further inclusion body development [56,57,58].

Several recent studies have proposed pathological α-syn to behave in a prion-like manner. Prions are classified based on their transmissibility as well as their ability to interact with and convert healthy proteins (PrP^C^) into the pathological form (PrP^SC^) [59,60]. Pathological α-syn displays many of these characteristics. The NAC region of α-syn contributes to stabilizing the β-sheet conformational change [2,61,62]. α-Syn fibrils have been shown to be insoluble, and persist in the extracellular environment, thus aiding transmissibility [44]. Additionally, recent work has shown that certain α-syn species can operate in a prion-like manner to auto-catalyze misfolding and aggregation, thereby resulting in neurotoxicity and neurodegeneration [60]. Radford et al. (2015) reported glial inclusion body formation in mice injected with dispersed GCI material [63]. Recasens et al. (2014) showed that human LB enriched fractions were able to induce PD-like pathology in macaque monkeys [64]. Rey et al. (2016) also demonstrated trans-neuronal transport in mice injected with α-syn [58]. Prusiner et al. (2015) discovered that MSA CNS homogenates can cause misfolding, inclusion formation and neurodegeneration in mice expressing the human familial PD α-syn mutant A53T, leading to MSA being termed a prion-like disease [65]. This discovery led to speculation that α-syn in MSA is of a different strain than that of PD or DLB due to the presentation of prion characteristics with the inoculation of MSA homogenates and the absence of these with PD and DLB homogenates. This may also explain the presence of inclusion bodies in and degeneration of oligodendrocytes observed in MSA. Curiously, aggregated α-syn was observed primarily in neurons rather than reproducing the mostly glial inclusions observed in MSA tissue [66]. Interestingly, homogenates from PD and DLB patients failed to induce significant neurodegeneration or α-syn pathology in the same study. This raises in question the identity of the pathogenic α-syn biochemical species in the different α-synucleinopathies and whether other factors, such as proteins, lipids and nucleic acids, in conjunction with α-syn are required for it to become prion-like.

Evidence for α-syn spread has also been observed in PD cases who received tissue grafts. Li et al. (2008) was the first reported case in which two subjects with PD developed LB pathology in grafted cells over a decade following the procedure [67]. Likewise, Kordower et al. (2008) revealed a similar result, in which an individual developed LBs in engrafted striatal neurons 14 years following tissue graft [68]. Ahn et al. (2012) reported similar findings in which fetal grafts developed PD pathology in addition to gliosis [69]. These results give further credence to the proposition that pathological α-syn can transmit to healthy cells and promote further α-syn aggregation similar to prion proteins. These studies highlight the potential for extracellular α-syn to be taken up by CNS cells and cause neurodegeneration in a prion-like manner. Although routes of α-syn spread and uptake are still poorly understood, recent studies have begun to clarify how this may occur. The current review will discuss potential routes of pathological α-syn spread, including exosomal transport, tunneling nanotubes, microglia and the glymphatic system.

## 4. Extracellular α-Syn, Secretion and Uptake

Several studies have demonstrated the existence of α-syn in the extracellular environment. El-Agnaf et al. (2006) demonstrated higher levels of oligomeric α-syn in the cerebrospinal fluid (CSF) and blood plasma of PD and DLB patients compared to healthy patients, suggesting that this may be due to release of the protein via cell death or other systems [70]. Later works have shown trans-synaptic release of oligomeric α-syn at neuron terminals mediated by Hsp70, later spreading to neighboring neurons, following the Braak model of spread and possibly explaining the spread of LBs in grafted neurons [71]. This was expanded upon by Fontaine et al., showing that Hsp70 in conjunction with its co-chaperone DnaJ also regulates the release of α-syn from the synapse [72]. This suggests that systems exist within the CNS to control and combat formation and spread of pathological α-syn oligomers, and that these regulatory systems are overloaded or non-functional in α-synucleinopathies. Peelaerts et al. (2015) explored the efficiency of extracellular spread of different α-syn strains comprising of oligomers, fibrils and ribbons in vivo, revealing that oligomers spread most efficiently [53]. In addition, dopaminergic neurons were observed to take up and enact trans-synaptic transport of all α-syn types. Whilst intercellular free α-syn movement may explain neuron–neuron spread to neighboring neurons, it cannot explain the occurrence of GCIs in MSA, as oligodendrocytes are not connected to neuronal synapses, or cases where α-syn pathology occurs in disconnected brain regions.

Neurons have another secretory mechanism at their disposal allowing for the release of cargo in the form of exosomes. Exosomes are 50–100 nm vesicles that facilitate intercellular communication by transporting specific proteins (e.g., heat shock proteins) or RNA (e.g., mRNA and miRNA) [73,74,75]. Composed of raft lipids such as cholesterol, exosome formation can occur in one of two ways. They can be directly shed from the plasma membrane, in which the cellular membrane forms a small vesicular bubble on its extracellular surface with the cargo inside before budding off the cell [76]. Alternatively, exosomes can be formed inside the multi-vesicular body, which then fuses with the cell membrane in an exocytic fashion thereby releasing cargo vesicles into the extracellular environment [77]. An example of this is oligodendrocytes releasing neuroprotective exosomes which support neuronal metabolism once internalized [78,79,80]. This process is not exclusive to oligodendrocytes as astrocytes and neurons facilitate bi-directional transfer of mitochondria in order to support neuronal homeostasis via micro-vesicles [81,82]. Other actions of exosomes include toxic material export as well as their potential role as a mechanism of intercellular α-syn spread.

As discussed previously, the normal function of α-syn is thought to be related to vesicle recycling within the presynaptic terminal involving direct interaction with lipids, whereas, neuronal exosomes are produced via multivesicular body-plasma membrane fusion or shedding of the neuronal membrane at the synapse [83]. Work by Danzer et al., revealed that α-syn oligomers can utilize exosomes in cell lines as well as primary neuronal cells and that not only do host cells expel α-syn oligomers via exosomes, but that α-syn within exosomes is more readily taken up via endocytosis than α-syn fibrils [84]. Oligomers occurred both on the inner and outer membrane of the expelled exosomes and dysfunction of autophagy reduced degradation of oligomers and increased exosomal release [84,85]. Calcium has also been implicated in exosomal α-syn release in PD, with increased calcium leading to the increased release of oligomer-containing exosomes [86]. As described previously, the oligodendroglial α-syn deposits observed in MSA are likely derived from an external source. Selective accumulation of α-syn occurred in primary astrocyte/oligodendrocyte co-cultures treated with α-syn protein [63] and cargo transport between neurons and oligodendrocytes and astrocytes has been demonstrated [87]. α-Syn was also found in vesicular structures (40 nm) in astrocytic endfeet of post-mortem MSA patient tissue with long disease duration, while α-syn pathology is observed both in astrocytes and Bergmann glia in α-synucleinopathies [88,89,90].

**Figure 1:**
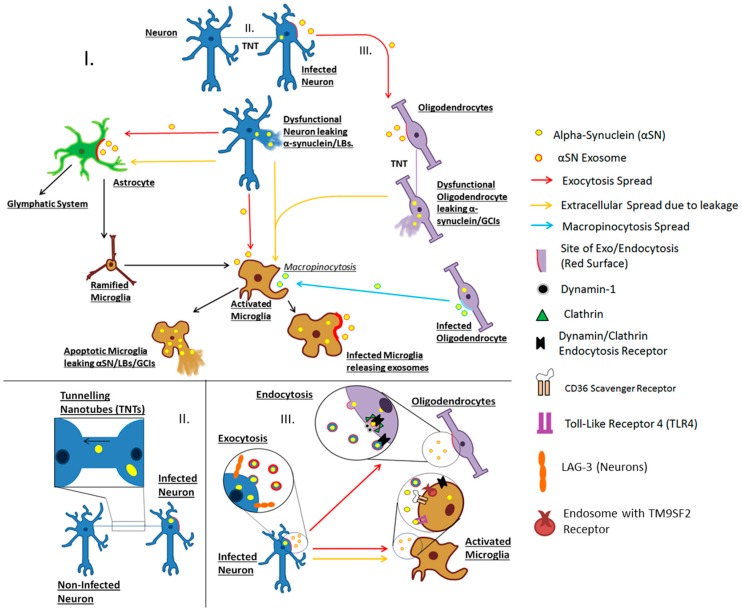
Hypothetical modes by which α-synuclein spreads in α-synucleinopathy disease. **I**. Release of α-synuclein/LBs via either exocytosis or membrane leakage due to apoptosis, necrosis or other damage. Astrocytes detect α-synuclein/LBs and signal for microglial recruitment by inflammatory factors. This also has the effect of activating microglia from the surveying ramified state to the phagocytic amoeboid phenotype (**black arrow**). Activation is also caused when ramified microglia detect α-synuclein either in exosomes or free in the extracellular matrix. Astrocytic activation can lead to dysregulation of glymphatic circulation via loss of aquaporin-4 polarization to endfeet, causing turbulent flow. Oligodendrocytes can take up α-synuclein-containing exosomes from neurons via endocytosis. Oligodendrocytes transfer α-syn to other oligodendrocytes either via exocytosis or tunneling-nanotubes (TNTs), eventually releasing α-synuclein through apoptosis/necrosis for phagocytosis by microglia. Oligodendrocytes may also release α-syn in exosomes. Microglia can engulf exosomes via macropinocytosis. Microglia perform phagocytosis on free and exosome-associated α-synuclein. Microglia may then mediate α-syn spread either via exosomes or via cell death or membrane leakage; **II**. F-actin TNTs exchange material between cells. In this illustration, an aggregate-bearing neuron (**right**) is transferring α-synuclein/LBs to an adjacent neuron (**left**) via a thick TNT causing spread of the pathological protein/inclusion body; and **III**. Transportation of α-synuclein via exo- and endocytosis. A neuron (**blue**), is undergoing exocytosis and the exosomes are travelling toward the oligodendrocyte (**purple**). Once close to the cell membrane of the oligodendrocyte, the exosomes may undergo different modes of endocytosis. One mode (**top**) involves fusing with the membrane of a target cell leading to the transfer of cargo. An alternate mode involves dynamin and clathrin mediated endocytosis, whereby the exosome binds to the receptor (**black**) which triggers its entry. Clathrin (**green triangles**) surrounds the budding vesicle whilst dynamin (**grey circles**) pinches the membrane off. The vesicle will then be fused with lysosomes for degradation. Microglia are shown activating clathrin/dynamin mediated endocytosis as well as microglial activation by CD36 scavenger receptor and Toll-like receptors (TLRs). Neurons have LAG3 and TM9SF2 receptors on the surface which when bound by fibrillar α-syn, mediate clathrin-dependent endocytosis.

How α-syn enters target cells following exocytosis is still largely unknown. Uptake via clathrin/dynamin-1 mediated endocytosis has been shown to occur in neurons, oligodendrocytes and microglia in vitro (Figure 1). However, inhibition of this process in dynamin-deficient cells or by blocking GTPases associated with this pathway did not completely inhibit α-syn entry [91,92,93,94,95]. Recently Mao et al. (2016) discovered that lymphocyte activating gene-3 (LAG-3), a leukocyte immunoglobulin protein also found on the surface of neurons, may potentiate the entry of fibrillar α-syn via clathrin-dependent endocytosis [96]. The results also show that LAG-3 is an α-syn-specific binding site, with tau and amyloid proteins found to bind to it in a non-specific manner. Based on the work by Mao et al. (2016) LAG3 appears to be expressed exclusively on neurons in the brain, however this does not rule out the participation of similar immunoglobulin proteins on astrocytic, microglial or oligodendroglial surfaces. Recently TM9SF2, a nonaspanin transmembrane protein found on endosomes, was implicated in α-syn spread and entry [97,98]. As endosomes deliver molecules to the lysosome for degradation, endocytosis of α-syn oligomers may in turn provide a link to impaired lysosomal degradation. Indeed, a variety of genetic factors linked to α-synucleinopathy are associated with the ineffective lysosomal degradation of α-syn [99,100].

Macropinocytosis is an actin-dependent mechanism whereby membrane ruffling allows entry of material into a variety of cell types in vitro and can be utilized by immune cells, such as microglia, to initiate lysosomal degradation. Heparan sulfate proteoglycans on the surface of cells have been shown to mediate the entry of α-syn fibrils via macropinocytosis [101]. Overall, current studies indicate that multiple mechanisms of exogenous α-syn cell entry exist. Further research is required to determine if other potential endocytic sites or receptors exist to facilitate α-syn entry into cells and whether therapeutic interventions can be developed to selectively inhibit uptake by healthy target cells.

## 5. Tunneling Nanotubes

Tunneling nanotubes (TNTs) are another mode of intercellular transfer, where F-actin interacts with the plasma membrane to form thin protrusions. Upon contacting and fusing with neighboring cells, TNTs function as a conduit to facilitate intercellular exchange and communication, with connections lasting up to several hours [102,103,104]. Currently, there are two classifications of TNTs described based on their length and thickness, each utilized by cells for different purposes. Thinner TNTs, composed solely of F-actin, are up to 15–60 μm long with a diameter of 50–200 nm thought to be used for bacterial and small molecule exchange [102,104,105]. Thicker TNTs composed of microtubules in addition to F-actin, can have a length of 30–140 µm with a diameter of >700 nm accommodating the transportation of larger cargo, such as mitochondria, endosomes and vesicles [102,104,106,107]. TNTs have been found between a variety of CNS cells such as neurons and astrocytes and in dendritic cells in vitro [106,108,109]. Sun et al., demonstrated TNTs between neurons and astrocytes, demonstrating that TNTs are not restricted to facilitating transport between cells of the same type [110].

There is evidence that pathogens, such as bacteria, HIV and the prion protein (PrP), have the ability to utilize TNTs to propagate into neighboring cells from an infected host in vitro, the spread of which could occur between cells of both similar and dissimilar cell type [105,106]. Recently, fibrillar α-syn was shown to be transported into lysosomes via TNTs in neuronal-like cells in vitro. This fibrillar α-syn was found to induce the subsequent aggregation of cytosolic α-syn in the target cells [102]. As TNTs are able to facilitate material exchange between differing cell types, it may be possible that TNTs could also mediate the spread of α-syn between different cell types in α-synucleinopathies, such as in MSA. Further investigation is required to elucidate the role of TNTs in intercellular α-syn transfer in α-synucleinopathies, although the small size and fragile nature of TNTs make their study in vitro and particularly in vivo technically challenging [111].

## 6. Microglia

Microglia, the principal immune-phagocytic cells of the CNS, display some characteristics which make them a potential vehicle of α-syn intercellular spread [112]. Under normal conditions, microglia exist in a resting state in non-overlapping domains across the brain where they survey for harmful or pathological molecules/proteins, the detection of which causes a change from a resting to a phagocytic state [113]. This change is accompanied by a morphological change from ramified to amoebic, allowing microglia to perform phagocytosis for the breakdown of harmful agents [113,114]. This activation is typically reinforced by the release of pro-inflammatory factors, such as interleukin-1β and tumor necrosis factor-α, for the recruitment of additional microglia to the site to allow for effective clearance [115].

Gliosis, the term used to describe the activation of glial cells such as microglia and astrocytes, is a major pathological feature of MSA and to a lesser extent DLB and PD. Elevated microglial activation in areas corresponding to α-syn deposition and neurodegeneration has been observed in patients with PD, DLB and MSA via positron emission tomography studies using a ligand to target activated macrophages and has been replicated in neuropathological studies of α-synucleinopathies [116,117]. Whether this is just a correlate of neurodegeneration or actively contributes to disease pathogenesis is currently unknown, although there is some evidence to show that microglia may have an active role in α-synucleinopathies. Increased pro-inflammatory cytokines are observed in MSA cases [22,118,119]. Activation of microglia is observed in PD, however the inflammatory response is not as widespread as in MSA [120,121]. Gliosis is also observed in DLB, and whilst gliosis also occurs in Alzheimer’s disease, it can be distinguished based on areas affected [122]. This difference in inflammatory profile may be related to different strains of α-syn as discussed earlier. Peelaerts et al., 2015 partially support this hypothesis, observing microglial activation to be most pronounced when exposed to fibrillar α-syn diffusing via the blood–brain barrier [53].

Microglia have been observed in vitro to take up exosomes released by oligodendrocytes containing α-syn via macropinocytosis, but lack the ability to effectively degrade fibrillar α-syn [78]. This inability to clear α-syn may be associated with impairment of the lysosomal pathway, the dominant mechanism associated with degradation after phagocytosis of α-syn and other pathological molecules. Previous studies have shown that lysosomal impairment is observed in PD, in both sporadic and familial cases, although this work has focused on neurons [85,123]. However, vaccination of transgenic mice was found to promote microglial clearance of α-syn [124].

α-Syn has also been found to utilize GM1-dependent lipid rafts by binding to the ganglioside to initiate receptor-mediated endocytosis in addition to macropinocytosis and clathrin/dynamin to enter microglia [93,94,125]. Other studies have shown that lipid rafts may mediate the localization of α-syn at the synapse [126,127,128]. This may suggest another α-syn entrance pathway into cells in α-synucleinopathies relevant to neurons and microglial. α-Syn also functions as a chemo-attractant, recruiting microglia to damaged cells [129]. Microglia are particularly sensitive to exogenous α-syn and it has been found to elicit pro-inflammatory and phagocytic responses in vitro via Toll-like receptor (TLR)-dependent pathways, such as TLR4, in addition to scavenger receptors, such as CD36 [63,130,131,132,133,134,135,136,137]. It is conceivable that α-syn could attract and activate microglia which fail to completely clear α-syn and potentially cause neurodegeneration themselves through an excessive pro-inflammatory response [135].

Microglia have been shown to play an active role in the progression of multiple neurodegenerative diseases. Specifically, depleting microglia and blocking the synthesis of exosomes in mice has been shown to decrease tau deposition in nearby healthy cells [136]. This suggests a potential synergy between microglia and exosomes in pathological spread in neurodegenerative conditions and is potentially an important mechanism of spread in α-synucleinopathies. In mice injected with pathological α-syn aggregates, microglial activation was observed to have spread further away from the site of injection over three weeks than the diffusion of α-syn aggregates [63]. Whilst there is little literature regarding microglia and their use of TNTs, TNTs or other systems may be employed for intercellular communication. Macrophages have been shown to utilize both forms of TNT for material exchange and phagocytic clearance of bacterial species between themselves and other connected cells [106,107]. Given the functional relationship between microglia and macrophages, it is possible that α-syn aggregates could use TNTs for intercellular spread to microglia. One important characteristic of microglial cells is their plasticity, allowing them to travel to distal brain regions. That plasticity, in combination with the inability of microglia to degrade pathological α-syn effectively, could make microglia an effective transport vehicle for α-syn during disease.

## 7. Glymphatic Clearance

There is evidence to suggest that the glymphatic system, facilitated by astrocytes, may also be involved as a mechanism of spread in α-synucleinopathies. The glymphatic system is analogous to the lymphatic system and operates to circulate nutrients and remove waste products via the exchange of interstitial fluid with cerebrospinal fluid (CSF). CSF flows into the perivascular space before being exchanged with interstitial fluid in the brain parenchyma. This process is facilitated by bulk flow due to pressure differentials and aquaporin-4 transporters on the endfeet of astrocytes which enclose the perivascular space [138]. While α-syn clearance by the glymphatic system has yet to be determined, other aggregation-prone proteins, such as amyloid β and tau, are cleared by the glymphatic system in models of Alzheimer’s disease and traumatic brain injury [139,140]. Additionally, phosphorylated α-syn has been found in the endfeet of astrocytes in a subset of MSA patients upon autopsy. While a recent study showed negative correlation between α-syn deposition and astrocytic aquaporin-4 expression in the temporal neocortex of PD patients [141]. This correlates with studies demonstrating that extracellular α-syn induces reactive astrogliosis [63]. Lower levels of α-syn in the CSF of patients with α-synucleinopathies compared to neurologically normal and Alzheimer’s patients has been observed which may indicate an impairment in α-syn clearance via the glymphatic system [142]. Investigating the clearance of α-syn via the glymphatic system in various in vivo models of α-synucleinopathies, combined with new functional glymphatic neuroimaging techniques in patients will help to assess any contributions of the glymphatic system to the pathophysiology occurring in α-synucleinopathies [143].

## 8. Summary and Concluding Remarks

Currently, exosome transport is the most widely investigated mode of α-syn spread. There are however still many factors relating to exosomal spread that are unknown, such as whether α-syn transport via exosomes is limited to neuron–neuron or if it is also utilized by other cell types. The multitude of routes of exosome-mediated cellular uptake renders therapeutic intervention a challenging target, whereby a candidate molecule would need to prevent entry via diverse mechanisms. Gaps in knowledge also exist regarding TNTs. Evidence of transportation has been shown to exist in vitro, however their role in α-synucleinopathies in vivo is still unclear. The role of the immune response of both astrocytes and microglia is also contentious whether this is primarily protective or degenerative. There is evidence of microglia propagating and enhancing the spread of tissue pathology in diseases such as Alzheimer’s disease, however further work on microglia is required concerning α-synucleinopathies.

Although multiple potential modes exist of α-syn spread in the brain, the pattern of spread in α-synucleinopathies is consistent between individual cases. Currently, α-syn spread according to the Braak staging scheme is predicted to occur between anatomically connected regions, forming a pattern based on α-syn spreading largely to neighboring regions. Yet vehicles such as exosomes and microglia have capabilities as long distance transporters to allow for spread to distal regions. In vivo, each of the mechanisms would be expected to occur either in stages or in concert. For example, gliosis involving increased activation of microglia and astrocytes may impair processes such as the glymphatic system. Thus, interactions between different modes of α-syn spread will further complicate successful therapeutic intervention, as multiple pathways may need to be targeted. Overall, future studies will need to investigate approaches to inhibiting the various potentially important mechanisms of α-syn spread in disease models which may identify novel targets for α-synucleinopathy therapies.

## Abbreviations

α-syn	α-Synuclein
CNS	Central Nervous System
CSF	Cerebrospinal Fluid
DLB	Dementia with Lewy Bodies
GCI	Glial Cytoplasmic Inclusions
MSA	Multiple System Atrophy
PD	Parkinson’s disease
TNTs	Tunneling Nanotubes
TLR	Toll-Like Receptor
LAG-3	Lymphocyte Activating Gene 3
LN	Lewy Neurite
NAC	Non-Aβ Component of Alzheimer’s Disease Amyloid

## References

[1] Barbour R., Kling K., Anderson J., Banducci K., Cole T., Diep L., Fox M., Goldstein J., Soriano F., Seubert P. (2008). Red Blood Cells Are the Major Source of α-Synuclein in Blood. Neurodegener. Dis..

[2] Theillet F., Binolfi A., Bekei B., Martorana A., Rose H., Stuiver M., Verzini S., Lorenz D., van Rossum M., Goldfarb D. (2016). Structural disorder of monomeric α-synuclein persists in mammalian cells. Nature.

[3] Weinreb P., Zhen W., Poon A., Conway K., Lansbury P. (1996). NACP, A Protein Implicated in Alzheimer’s Disease and Learning, Is Natively Unfolded. Biochemistry.

[4] Cheng F., Vivacqua G., Yu S. (2011). The role of α-synuclein in neurotransmission and synaptic plasticity. J. Chem. Neuroanat..

[5] Burre J., Sharma M., Tsetsenis T., Buchman V., Etherton M., Sudhof T. (2010). α-Synuclein Promotes SNARE-Complex Assembly in Vivo and in Vitro. Science.

[6] Perez R., Waymire J., Lin E., Liu J., Guo F., Zigmond M. (2002). A role for α-synuclein in the regulation of dopamine biosynthesis. J. Neurosci..

[7] Larsen K., Schmitz Y., Troyer M., Mosharov E., Dietrich P., Quazi A., Savalle M., Nemani V., Chaudhry F., Edwards R. (2006). α-Synuclein Overexpression in PC12 and Chromaffin Cells Impairs Catecholamine Release by Interfering with a Late Step in Exocytosis. J. Neurosci..

[8] Yavich L., Jäkälä P., Tanila H. (2006). Abnormal compartmentalization of norepinephrine in mouse dentate gyrus in α-synuclein knockout and A30P transgenic mice. J. Neurochem..

[9] Yavich L., Tanilla H., Vepsalainen S., Jakala P. (2004). Role of α-Synuclein in Presynaptic Dopamine Recruitment. J. Neurosci..

[10] Bartels T., Choi J., Selkoe D. (2011). α-Synuclein occurs physiologically as a helically folded tetramer that resists aggregation. Nature.

[11] Jakes R., Spillantini M., Goedert M. (1994). Identification of two distinct synucleins from human brain. FEBS Lett..

[12] Luth E., Bartels T., Dettmer U., Kim N., Selkoe D. (2015). Purification of α-Synuclein from Human Brain Reveals an Instability of Endogenous Multimers as the Protein Approaches Purity. Biochemistry.

[13] Burré J., Vivona S., Diao J., Sharma M., Brunger A., Südhof T. (2013). Properties of native brain α-synuclein. Nature.

[14] McCann H., Stevens C., Cartwright H., Halliday G. (2014). α-Synucleinopathy phenotypes. Park. Relat. Disord..

[15] Sveinbjornsdottir S. (2016). The clinical symptoms of Parkinson’s disease. J. Neurochem..

[16] Menza M., Robertson-Hoffman D., Bonapace A. (1993). Parkinson’s disease and anxiety: Comorbidity with depression. Biol. Psychiatry.

[17] McKeith I., Mintzer J., Aarsland D., Burn D., Chiu H., Cohen-Mansfield J., Dickson D., Dubois B., Duda J., Feldman H. (2004). Dementia with Lewy bodies. Lancet Neurol..

[18] Longo D., Fanciulli A., Wenning G. (2015). Multiple-System Atrophy. N. Engl. J. Med..

[19] Ubhi K., Low P., Masliah E. (2011). Multiple system atrophy: A clinical and neuropathological perspective. Trends Neurosci..

[20] Braak H., Tredici K., Rüb U., de Vos R., Jansen Steur E., Braak E. (2003). Staging of brain pathology related to sporadic Parkinson’s disease. Neurobiol. Aging.

[21] Marttila R., Rhine U. (1991). Progression and survival in Parkinson’s disease. Acta Neurol..

[22] Wenning G., Stefanova N. (2009). Recent developments in multiple system atrophy. J. Neurol..

[23] Williams M., Xiong C., Morris J., Galvin J. (2006). Survival and mortality differences between dementia with Lewy bodies vs. Alzheimer disease. Neurology.

[24] Spillantini M., Schmidt M., Lee V., Trojanowski J., Jakes R., Goedert M. (1997). α-Synuclein in Lewy bodies. Nature.

[25] Engelender S. (2008). Ubiquitination of α-synuclein and autophagy in Parkinson’s disease. Autophagy.

[26] Braak H., Sandmann-Keil D., Gai W., Braak E. (1999). Extensive axonal Lewy neurites in Parkinson’s disease: A novel pathological feature revealed by α-synuclein immunocytochemistry. Neurosci. Lett..

[27] Galloway P., Mulvihill P., Perry G. (1992). Filaments of Lewy bodies contain insoluble cytoskeletal elements. Am. J. Pathol..

[28] Kanazawa T., Uchihara T., Takahashi A., Nakamura A., Orimo S., Mizusawa H. (2008). Three-Layered Structure Shared Between Lewy Bodies and Lewy Neurites—Three-Dimensional Reconstruction of Triple-Labeled Sections. Brain Pathol..

[29] Miki Y., Mori F., Tanji K., Wakabayashi K. (2010). Athology of neuro-glial α-synucleinopathy (Lewy body disease and multiple system atrophy). Hirosaki Med. J..

[30] Tu P., Galvin J., Baba M., Giasson B., Tomita T., Leight S., Nakajo S., Iwatsubo T., Trojanowski J., Lee V. (1998). Glial cytoplasmic inclusions in white matter oligodendrocytes of multiple system atrophy brains contain insoluble α-synuclein. Ann. Neurol..

[31] Grazia Spillantini M., Anthony Crowther R., Jakes R., Cairns N., Lantos P., Goedert M. (1998). Filamentous α-synuclein inclusions link multiple system atrophy with Parkinson’s disease and dementia with Lewy bodies. Neurosci. Lett..

[32] Ahmed Z., Asi Y., Sailer A., Lees A., Houlden H., Revesz T., Holton J. (2012). The neuropathology, pathophysiology and genetics of multiple system atrophy. Neuropathol. Appl. Neurobiol..

[33] Asi Y., Simpson J., Heath P., Wharton S., Lees A., Revesz T., Houlden H., Holton J. (2014). α-Synuclein mRNA expression in oligodendrocytes in MSA. Glia.

[34] Djelloul M., Holmqvist S., Boza-Serrano A., Azevedo C., Yeung M., Goldwurm S., Frisén J., Deierborg T., Roybon L. (2015). α-Synuclein Expression in the Oligodendrocyte Lineage: An In Vitro and In Vivo Study Using Rodent and Human Models. Stem Cell Rep..

[35] Lesage S., Anheim M., Letournel F., Bousset L., Honoré A., Rozas N., Pieri L., Madiona K., Dürr A., Melki R. (2013). G51D α-synuclein mutation causes a novel Parkinsonian-pyramidal syndrome. Ann. Neurol..

[36] Polymeropoulos M. (1997). Mutation in the α-Synuclein Gene Identified in Families with Parkinson’s Disease. Science.

[37] Chartier-Harlin M., Kachergus J., Roumier C., Mouroux V., Douay X., Lincoln S., Levecque C., Larvor L., Andrieux J., Hulihan M. (2004). α-Synuclein locus duplication as a cause of familial Parkinson’s disease. Lancet.

[38] Simón-Sánchez J., Schulte C., Bras J., Sharma M., Gibbs J., Berg D., Paisan-Ruiz C., Lichtner P., Scholz S., Hernandez D. (2009). Genome-wide association study reveals genetic risk underlying Parkinson’s disease. Nat. Genet..

[39] Nalls M., Pankratz N., Lill C., Do C., Hernandez D., Saad M., DeStefano A., Kara E., Bras J., Sharma M. (2014). Large-scale meta-analysis of genome-wide association data identifies six new risk loci for Parkinson’s disease. Nat. Genet..

[40] Satake W., Nakabayashi Y., Mizuta I., Hirota Y., Ito C., Kubo M., Kawaguchi T., Tsunoda T., Watanabe M., Takeda A. (2009). Genome-wide association study identifies common variants at four loci as genetic risk factors for Parkinson’s disease. Nat. Genet..

[41] Rcom-H’cheo-Gauthier A., Osborne S., Meedeniya A., Pountney D. (2016). Calcium: α-Synuclein Interactions in α-Synucleinopathies. Front. Neurosci..

[42] Fujiwara H., Hasegawa M., Dohmae N., Kawashima A., Masliah E., Goldberg M., Shen J., Takio K., Iwatsubo T. (2002). α-Synuclein is phosphorylated in synucleinopathy lesions. Nat. Cell Biol..

[43] Reynolds A., Glanzer J., Kadiu I., Ricardo-Dukelow M., Chaudhuri A., Ciborowski P., Cerny R., Gelman B., Thomas M., Mosley R. (2007). Nitrated α-synuclein-activated microglial profiling for Parkinson’s disease. J. Neurochem..

[44] Cookson M. (2009). α-Synuclein and neuronal cell death. Mol. Neurodegener..

[45] Anderson J., Walker D., Goldstein J., de Laat R., Banducci K., Caccavello R., Barbour R., Huang J., Kling K., Lee M. (2006). Phosphorylation of Ser-129 Is the Dominant Pathological Modification of α-Synuclein in Familial and Sporadic Lewy Body Disease. J. Biol. Chem..

[46] Ueda K., Fukushima H., Masliah E., Xia Y., Iwai A., Yoshimoto M., Otero D., Kondo J., Ihara Y., Saitoh T. (1993). Molecular cloning of cDNA encoding an unrecognized component of amyloid in Alzheimer disease. Proc. Natl. Acad. Sci. USA.

[47] Bousset L., Pieri L., Ruiz-Arlandis G., Gath J., Jensen P., Habenstein B., Madiona K., Olieric V., Böckmann A., Meier B. (2013). Structural and functional characterization of two α-synuclein strains. Nat. Commun..

[48] Gai W., Pountney D., Power J., Li Q., Culvenor J., McLean C., Jensen P., Blumbergs P. (2003). α-Synuclein fibrils constitute the central core of oligodendroglial inclusion filaments in multiple system atrophy. Exp. Neurol..

[49] Pountney D., Lowe R., Quilty M., Vickers J., Voelcker N., Gai W. (2004). Annular α-synuclein species from purified multiple system atrophy inclusions. J. Neurochem..

[50] Paviour D., Price S., Lees A., Fox N. (2007). MRI derived brain atrophy in PSP and MSA-P. J. Neurol..

[51] Brettschneider J., Irwin D., Boluda S., Byrne M., Fang L., Lee E., Robinson J., Suh E., van Deerlin V., Toledo J. (2016). Progression of α-synuclein pathology in multiple system atrophy of the cerebellar type. Neuropathol. Appl. Neurobiol..

[52] McKeith I., Dickson D., Lowe J., Emre M., O’Brien J., Feldman H., Cummings J., Duda J., Lippa C., Perry E. (2005). Diagnosis and management of dementia with Lewy bodies: Third report of the DLB consortium. Neurology.

[53] Peelaerts W., Bousset L., van der Perren A., Moskalyuk A., Pulizzi R., Giugliano M., van den Haute C., Melki R., Baekelandt V. (2015). α-Synuclein strains cause distinct synucleinopathies after local and systemic administration. Nature.

[54] Luk K., Kehm V., Carroll J., Zhang B., O’Brien P., Trojanowski J., Lee V. (2012). Pathological α-Synuclein Transmission Initiates Parkinson-like Neurodegeneration in Nontransgenic Mice. Science.

[55] Osterberg V., Spinelli K., Weston L., Luk K., Woltjer R., Unni V. (2015). Progressive Aggregation of α-Synuclein and Selective Degeneration of Lewy Inclusion-Bearing Neurons in a Mouse Model of Parkinsonism. Cell Rep..

[56] Paumier K., Luk K., Manfredsson F., Kanaan N., Lipton J., Collier T., Steece-Collier K., Kemp C., Celano S., Schulz E. (2015). Intrastriatal injection of pre-formed mouse α-synuclein fibrils into rats triggers α-synuclein pathology and bilateral nigrostriatal degeneration. Neurobiol. Dis..

[57] Sacino A., Brooks M., McGarvey N., McKinney A., Thomas M., Levites Y., Ran Y., Golde T., Giasson B. (2013). Induction of CNS α-synuclein pathology by fibrillar and non-amyloidogenic recombinant α-synuclein. Acta Neuropathol. Commun..

[58] Rey N.L., Steiner J.A., Maroof N., Luk K.C., Madaj Z., Trojanowski J.Q., Lee V.M., Brundin P. (2016). Widespread transneuronal propagation of α-synucleinopathy triggered in olfactory bulb mimics prodromal Parkinson’s disease. J. Exp. Med..

[59] Kovacs G., Budka H. (2008). Prion Diseases: From Protein to Cell Pathology. Am. J. Pathol..

[60] Goedert M. (2015). Alzheimer’s and Parkinson’s diseases: The prion concept in relation to assembled Aβ, tau, and α-synuclein. Science.

[61] Iwai A. (2000). Properties of NACP/α-synuclein and its role in Alzheimer’s disease. Biochim. Biophys. Acta Mol. Basis Dis..

[62] Rodriguez J., Ivanova M., Sawaya M., Cascio D., Reyes F., Shi D., Sangwan S., Guenther E., Johnson L., Zhang M. (2015). Structure of the toxic core of α-synuclein from invisible crystals. Nature.

[63] Radford R., Rcom-H’cheo-Gauthier A., Wong M.B., Eaton E.D., Quilty M., Blizzard C., Norazit A., Meedeniya A., Vickers J.C., Gai W.P. (2015). The degree of astrocyte activation in multiple system atrophy is inversely proportional to the distance to α-synuclein inclusions. Mol. Cell Neurosci..

[64] Recasens A., Dehay B., Bové J., Carballo-Carbajal I., Dovero S., Pérez-Villalba A., Fernagut P., Blesa J., Parent A., Perier C. (2014). Lewy body extracts from Parkinson disease brains trigger α-synuclein pathology and neurodegeneration in mice and monkeys. Ann. Neurol..

[65] Prusiner S., Woerman A., Mordes D., Watts J., Rampersaud R., Berry D., Patel S., Oehler A., Lowe J., Kravitz S. (2015). Evidence for α-synuclein prions causing multiple system atrophy in humans with parkinsonism. Proc. Natl. Acad. Sci. USA.

[66] Cykowski M., Coon E., Powell S., Jenkins S., Benarroch E., Low P., Schmeichel A., Parisi J. (2015). Expanding the spectrum of neuronal pathology in multiple system atrophy. Brain.

[67] Li J., Englund E., Holton J., Soulet D., Hagell P., Lees A., Lashley T., Quinn N., Rehncrona S., Björklund A. (2008). Lewy bodies in grafted neurons in subjects with Parkinson’s disease suggest host-to-graft disease propagation. Nat. Med..

[68] Kordower J., Chu Y., Hauser R., Freeman T., Olanow C. (2008). Lewy body–like pathology in long-term embryonic nigral transplants in Parkinson’s disease. Nat. Med..

[69] Ahn T., Langston J., Aachi V., Dickson D. (2012). Relationship of neighboring tissue and gliosis to α-synuclein pathology in a fetal transplant for Parkinson’s disease. Am. J. Neurodegener. Dis..

[70] El-Agnaf O., Salem S., Paleologou K., Curran M., Gibson M., Court J., Schlossmacher M., Allsop D. (2006). Detection of oligomeric forms of α-synuclein protein in human plasma as a potential biomarker for Parkinson’s disease. FASEB J..

[71] Danzer K., Ruf W., Putcha P., Joyner D., Hashimoto T., Glabe C., Hyman B., McLean P. (2011). Heat-shock protein 70 modulates toxic extracellular α-synuclein oligomers and rescues trans-synaptic toxicity. FASEB J..

[72] Fontaine S., Zheng D., Sabbagh J., Martin M., Chaput D., Darling A., Trotter J., Stothert A., Nordhues B., Lussier A. (2016). DnaJ/Hsc70 chaperone complexes control the extracellular release of neurodegenerative-associated proteins. EMBO J..

[73] Valadi H., Ekström K., Bossios A., Sjöstrand M., Lee J., Lötvall J. (2007). Exosome-mediated transfer of mRNAs and microRNAs is a novel mechanism of genetic exchange between cells. Nat. Cell Biol..

[74] Gibbings D., Ciaudo C., Erhardt M., Voinnet O. (2009). Multivesicular bodies associate with components of miRNA effector complexes and modulate miRNA activity. Nat. Cell Biol..

[75] Lancaster G., Febbraio M. (2005). Exosome-dependent Trafficking of HSP70: A novel secretory pathway for cellular stress proteins. J. Biol. Chem..

[76] Beaudoin A., Grondin G. (1991). Shedding of vesicular material from the cell surface of eukaryotic cells: Different cellular phenomena. Biochim. Biophys. Acta Rev. Biomembr..

[77] Denzer K., Kleijmeer M., Heijnen H., Stoorvogel W., Geuze H. (2000). Exosome: From internal vesicle of the multivesicular body to intercellular signaling device. J. Cell Sci..

[78] Fitzner D., Schnaars M., van Rossum D., Krishnamoorthy G., Dibaj P., Bakhti M., Regen T., Hanisch U., Simons M. (2011). Selective transfer of exosomes from oligodendrocytes to microglia by macropinocytosis. J. Cell Sci..

[79] Frühbeis C., Fröhlich D., Kuo W., Krämer-Albers E. (2013). Extracellular vesicles as mediators of neuron-glia communication. Front. Cell. Neurosci..

[80] Frühbeis C., Fröhlich D., Kuo W., Amphornrat J., Thilemann S., Saab A., Kirchhoff F., Möbius W., Goebbels S., Nave K. (2013). Neurotransmitter-Triggered Transfer of Exosomes Mediates Oligodendrocyte–Neuron Communication. PLoS Biol..

[81] Davis C., Kim K., Bushong E., Mills E., Boassa D., Shih T., Kinebuchi M., Phan S., Zhou Y., Bihlmeyer N. (2014). Transcellular degradation of axonal mitochondria. Proc. Natl. Acad. Sci. USA.

[82] Hayakawa K., Esposito E., Wang X., Terasaki Y., Liu Y., Xing C., Ji X., Lo E. (2016). Transfer of mitochondria from astrocytes to neurons after stroke. Nature.

[83] Von Bartheld C., Altick A. (2011). Multivesicular bodies in neurons: Distribution, protein content, and trafficking functions. Prog. Neurobiol..

[84] Danzer K., Kranich L., Ruf W., Cagsal-Getkin O., Winslow A., Zhu L., Vanderburg C., McLean P. (2012). Exosomal cell-to-cell transmission of α synuclein oligomers. Mol. Neurodegener..

[85] Alvarez-Erviti L., Seow Y., Schapira A., Gardiner C., Sargent I., Wood M., Cooper J. (2011). Lysosomal dysfunction increases exosome-mediated α-synuclein release and transmission. Neurobiol. Dis..

[86] Emmanouilidou E., Melachroinou K., Roumeliotis T., Garbis S., Ntzouni M., Margaritis L., Stefanis L., Vekrellis K. (2010). Cell-Produced-Synuclein Is Secreted in a Calcium-Dependent Manner by Exosomes and Impacts Neuronal Survival. J. Neurosci..

[87] Frohlich D., Kuo W., Fruhbeis C., Sun J., Zehendner C., Luhmann H., Pinto S., Toedling J., Trotter J., Kramer-Albers E. (2014). Multifaceted effects of oligodendroglial exosomes on neurons: Impact on neuronal firing rate, signal transduction and gene regulation. Philos. Trans. R. Soc. B Biol. Sci..

[88] Nakamura K., Mori F., Kon T., Tanji K., Miki Y., Tomiyama M., Kurotaki H., Toyoshima Y., Kakita A., Takahashi H. (2016). Accumulation of phosphorylated α-synuclein in subpial and periventricular astrocytes in multiple system atrophy of long duration. Neuropathology.

[89] Braak H., Sastre M., Del Tredici K. (2007). Development of α-synuclein immunoreactive astrocytes in the forebrain parallels stages of intraneuronal pathology in sporadic Parkinson’s disease. Acta Neuropathol..

[90] Piao Y., Mori F., Hayashi S., Tanji K., Yoshimoto M., Kakita A., Wakabayashi K., Takahashi H. (2003). α-Synuclein pathology affecting Bergmann glia of the cerebellum in patients with α-synucleinopathies. Acta Neuropathol..

[91] Reyes J., Rey N., Bousset L., Melki R., Brundin P., Angot E. (2013). α-Synuclein transfers from neurons to oligodendrocytes. Glia.

[92] Desplats P., Lee H., Bae E., Patrick C., Rockenstein E., Crews L., Spencer B., Masliah E., Lee S. (2009). Inclusion formation and neuronal cell death through neuron-to-neuron transmission of α-synuclein. Proc. Natl. Acad. Sci. USA.

[93] Liu J., Zhou Y., Wang Y., Fong H., Murray T., Zhang J. (2007). Identification of Proteins Involved in Microglial Endocytosis of α-Synuclein. J. Proteome Res..

[94] Lee H., Suk J., Bae E., Lee J., Paik S., Lee S. (2008). Assembly-dependent endocytosis and clearance of extracellular α-synuclein. Int. J. Biochem. Cell Biol..

[95] Konno M., Hasegawa T., Baba T., Miura E., Sugeno N., Kikuchi A., Fiesel F., Sasaki T., Aoki M., Itoyama Y. (2012). Suppression of dynamin GTPase decreases α-synuclein uptake by neuronal and oligodendroglial cells: A potent therapeutic target for synucleinopathy. Mol. Neurodegener..

[96] Mao X., Ou M., Karuppagounder S., Kam T., Yin X., Xiong Y., Ge P., Umanah G., Brahmachari S., Shin J. (2016). Pathological α-synuclein transmission initiated by binding lymphocyte-activation gene 3. Science.

[97] Schimmöller F., Díaz E., Mühlbauer B., Pfeffer S. (1998). Characterization of a 76kDa endosomal, multispanning membrane protein that is highly conserved throughout evolution. Gene.

[98] Wadman M. (2016). Rogue protein’s partners offer hope in Parkinson’s disease. Science.

[99] Usenovic M., Tresse E., Mazzulli J., Taylor J., Krainc D. (2012). Deficiency of ATP13A2 Leads to Lysosomal Dysfunction, α-Synuclein Accumulation, and Neurotoxicity. J. Neurosci..

[100] Siebert M., Sidransky E., Westbroek W. (2014). Glucocerebrosidase is shaking up the synucleinopathies. Brain.

[101] Holmes B., DeVos S., Kfoury N., Li M., Jacks R., Yanamandra K., Ouidja M., Brodsky F., Marasa J., Bagchi D. (2013). Heparan sulfate proteoglycans mediate internalization and propagation of specific proteopathic seeds. Proc. Natl. Acad. Sci. USA.

[102] Abounit S., Bousset L., Loria F., Zhu S., de Chaumont F., Pieri L., Olivo-Marin J., Melki R., Zurzolo C. (2016). Tunneling nanotubes spread fibrillar α-synuclein by intercellular trafficking of lysosomes. EMBO J..

[103] Agnati L., Fuxe K. (2014). Extracellular-vesicle type of volume transmission and tunnelling-nanotube type of wiring transmission add a new dimension to brain neuro-glial networks. Philos. Trans. R. Soc. B Biol. Sci..

[104] Gousset K., Schiff E., Langevin C., Marijanovic Z., Caputo A., Browman D., Chenouard N., de Chaumont F., Martino A., Enninga J. (2009). Prions hijack tunnelling nanotubes for intercellular spread. Nat. Cell Biol..

[105] Eugenin E., Gaskill P., Berman J. (2009). Tunneling nanotubes (TNT) are induced by HIV-infection of macrophages: A potential mechanism for intercellular HIV trafficking. Cell. Immunol..

[106] Onfelt B., Nedvetzki S., Yanagi K., Davis D. (2004). Cutting Edge: Membrane Nanotubes Connect Immune Cells. J. Immunol..

[107] Onfelt B., Nedvetzki S., Benninger R., Purbhoo M., Sowinski S., Hume A., Seabra M., Neil M., French P., Davis D. (2006). Structurally Distinct Membrane Nanotubes between Human Macrophages Support Long-Distance Vesicular Traffic or Surfing of Bacteria. J. Immunol..

[108] Zhu D., Tan K., Zhang X., Sun A., Sun G., Lee J. (2005). Hydrogen peroxide alters membrane and cytoskeleton properties and increases intercellular connections in astrocytes. J. Cell Sci..

[109] Rustom A., Saffrich R., Markovic I., Walther P., Gerdes H. (2004). Nanotubular Highways for Intercellular Organelle Transport. Science.

[110] Sun X., Wang Y., Zhang J., Tu J., Wang X., Su X., Wang L., Zhang Y. (2012). Tunneling-nanotube direction determination in neurons and astrocytes. Cell Death Dis..

[111] McCoy-Simandle K., Hanna S., Cox D. (2016). Exosomes and nanotubes: Control of immune cell communication. Int. J. Biochem. Cell Biol..

[112] Kettenmann H., Hanisch U., Noda M., Verkhratsky A. (2011). Physiology of Microglia. Physiol. Rev..

[113] Jonas R., Yuan T., Liang Y., Jonas J., Tay D., Ellis-Behnke R. (2012). The Spider Effect: Morphological and Orienting Classification of Microglia in Response to Stimuli in Vivo. PLoS ONE.

[114] Perry V., Cunningham C., Holmes C. (2007). Systemic infections and inflammation affect chronic neurodegeneration. Nat. Rev. Immunol..

[115] Neumann H., Kotter M., Franklin R. (2008). Debris clearance by microglia: An essential link between degeneration and regeneration. Brain.

[116] Fellner L., Stefanova N. (2012). The Role of Glia in α-Synucleinopathies. Mol. Neurobiol..

[117] Politis M., Su P., Piccini P. (2012). Imaging of microglia in patients with neurodegenerative disorders. Front. Pharmacol..

[118] Kaufman E., Hall S., Surova Y., Widner H., Hansson O., Lindqvist D. (2013). Proinflammatory Cytokines Are Elevated in Serum of Patients with Multiple System Atrophy. PLoS ONE.

[119] Koga S., Aoki N., Uitti R., van Gerpen J., Cheshire W., Josephs K., Wszolek Z., Langston J., Dickson D. (2015). When DLB, PD, and PSP masquerade as MSA. Neurology.

[120] Nagatsu T., Sawada M. (2005). Inflammatory Process in Parkinsons Disease: Role for Cytokines. Curr. Pharm. Des..

[121] Tufekci K., Meuwissen R., Genc S., Genc K. (2012). Inflammation in Parkinson’s Disease. Adv. Protein Chem. Struct. Biol..

[122] Higuchi M., Tashiro M., Arai H., Okamura N., Hara S., Higuchi S., Itoh M., Shin R., Trojanowski J., Sasaki H. (2000). Glucose Hypometabolism and Neuropathological Correlates in Brains of Dementia with Lewy Bodies. Exp. Neurol..

[123] Chu Y., Dodiya H., Aebischer P., Olanow C., Kordower J. (2009). Alterations in lysosomal and proteasomal markers in Parkinson’s disease: Relationship to α-synuclein inclusions. Neurobiol. Dis..

[124] Masliah E., Rockenstein E., Adame A., Alford M., Crews L., Hashimoto M., Seubert P., Lee M., Goldstein J., Chilicote T. (2005). Effects of α-synuclein immunization in a mouse model of Parkinson’s disease. Neuron.

[125] Park J., Kim K., Lee S., Ryu J., Chung K., Choo Y., Jou I., Kim J., Park S. (2009). On the mechanism of internalization of α-synuclein into microglia: Roles of ganglioside GM1 and lipid raft. J. Neurochem..

[126] Fortin D., Troyer M., Nakamura K., Kubo S., Anthony M., Edwards R. (2004). Lipid Rafts Mediate the Synaptic Localization of α-Synuclein. J. Neurosci..

[127] Bar-On P., Crews L., Koob A., Mizuno H., Adame A., Spencer B., Masliah E. (2008). Statins reduce neuronal α-synuclein aggregation in in vitro models of Parkinson’s disease. J. Neurochem..

[128] Kubo S., Nemani V., Chalkley R., Anthony M., Hattori N., Mizuno Y., Edwards R., Fortin D. (2005). A Combinatorial Code for the Interaction of α-Synuclein with Membranes. J. Biol. Chem..

[129] Wang S., Chu C., Stewart T., Ginghina C., Wang Y., Nie H., Guo M., Wilson B., Hong J., Zhang J. (2015). α-Synuclein, a chemoattractant, directs microglial migration via H_2_O_2_-dependent Lyn phosphorylation. Proc. Natl. Acad. Sci. USA.

[130] Su X., Maguire-Zeiss K., Giuliano R., Prifti L., Venkatesh K., Federoff H. (2008). Synuclein activates microglia in a model of Parkinson’s disease. Neurobiol. Aging.

[131] Stefanova N., Fellner L., Reindl M., Masliah E., Poewe W., Wenning G. (2011). Toll-Like Receptor 4 Promotes α-Synuclein Clearance and Survival of Nigral Dopaminergic Neurons. Am. J. Pathol..

[132] Kim C., Ho D., Suk J., You S., Michael S., Kang J., Joong Lee S., Masliah E., Hwang D., Lee H. (2013). Neuron-released oligomeric α-synuclein is an endogenous agonist of TLR2 for paracrine activation of microglia. Nat. Commun..

[133] Roodveldt C., Labrador-Garrido A., Gonzalez-Rey E., Lachaud C., Guilliams T., Fernandez-Montesinos R., Benitez-Rondan A., Robledo G., Hmadcha A., Delgado M. (2013). Preconditioning of Microglia by α-Synuclein Strongly Affects the Response Induced by Toll-like Receptor (TLR) Stimulation. PLoS ONE.

[134] Fellner L., Irschick R., Schanda K., Reindl M., Klimaschewski L., Poewe W., Wenning G., Stefanova N. (2013). Toll-like receptor 4 is required for α-synuclein dependent activation of microglia and astroglia. Glia.

[135] Tang Y., Le W. (2015). Differential Roles of M1 and M2 Microglia in Neurodegenerative Diseases. Mol. Neurobiol..

[136] Asai H., Ikezu S., Tsunoda S., Medalla M., Luebke J., Haydar T., Wolozin B., Butovsky O., Kügler S., Ikezu T. (2015). Depletion of microglia and inhibition of exosome synthesis halt tau propagation. Nat. Neurosci..

[137] Vieira B.D., Radford R.A., Chung R.S., Guillemin G.J., Pountney D.L. (2015). Neuroinflammation in Multiple System Atrophy: Response to and Cause of α-Synuclein Aggregation. Front. Cell Neurosci..

[138] Jessen N., Munk A., Lundgaard I., Nedergaard M. (2015). The Glymphatic System: A Beginner’s Guide. Neurochem. Res..

[139] Iliff J., Wang M., Liao Y., Plogg B., Peng W., Gundersen G., Benveniste H., Vates G., Deane R., Goldman S. (2012). A Paravascular Pathway Facilitates CSF Flow Through the Brain Parenchyma and the Clearance of Interstitial Solutes, Including Amyloid β. Sci. Transl. Med..

[140] Iliff J., Chen M., Plog B., Zeppenfeld D., Soltero M., Yang L., Singh I., Deane R., Nedergaard M. (2014). Impairment of Glymphatic Pathway Function Promotes Tau Pathology after Traumatic Brain Injury. J. Neurosci..

[141] Hoshi A., Tsunoda A., Tada M., Nishizawa M., Ugawa Y., Kakita A. (2016). Expression of aquaporin 1 and aquaporin 4 in the temporal neocortex of patients with Parkinson’s disease. Brain Pathol..

[142] Gao L., Tang H., Nie K., Wang L., Zhao J., Gan R., Huang J., Zhu R., Feng S., Duan Z. (2014). Cerebrospinal fluid α-synuclein as a biomarker for Parkinson’s disease diagnosis: A systematic review and meta-analysis. Int. J. Neurosci..

[143] Lundgaard I., Lu M., Yang E., Peng W., Mestre H., Hitomi E., Deane R., Nedergaard M. (2016). Glymphatic clearance controls state-dependent changes in brain lactate concentration. J. Cereb. Blood Flow Metab..

